# Longitudinal profiles of the fecal metabolome during the first 2 years of life

**DOI:** 10.1038/s41598-023-28862-z

**Published:** 2023-02-02

**Authors:** Elizabeth A. Holzhausen, Natalie Shen, Bridget Chalifour, ViLinh Tran, Zhenjiang Li, Jeremy A. Sarnat, Howard H. Chang, Dean P. Jones, Michael I. Goran, Donghai Liang, Tanya L. Alderete

**Affiliations:** 1 Department of Integrative Physiology, University of Colorado – Boulder, Boulder, CO USA; 2 Rollins School of Public Health, Emory University, Atlanta, GA USA; 3 School of Medicine, Emory University, Atlanta, GA USA; 4 Children’s Hospital Los Angeles, Los Angeles, CA USA

**Keywords:** Metabolism, Metabolomics

## Abstract

During the first 2 years of life, the infant gut microbiome is rapidly developing, and gut bacteria may impact host health through the production of metabolites that can have systemic effects. Thus, the fecal metabolome represents a functional readout of gut bacteria. Despite the important role that fecal metabolites may play in infant health, the development of the infant fecal metabolome has not yet been thoroughly characterized using frequent, repeated sampling during the first 2 years of life. Here, we described the development of the fecal metabolome in a cohort of 101 Latino infants with data collected at 1-, 6-, 12-, 18-, and 24-months of age. We showed that the fecal metabolome is highly conserved across time and highly personalized, with metabolic profiles being largely driven by intra-individual variability. Finally, we also identified several novel metabolites and metabolic pathways that changed significantly with infant age, such as valerobetaine and amino acid metabolism, among others.

## Introduction

For infants, the first 1000 days represent a critical developmental window, during which metabolic, endocrine, neural, and immune systems are rapidly maturing. In tandem, the infant gut microbiome is also rapidly developing, until 2–3 years of age, when it reaches a more adult-like configuration^1^. This early colonization of the infant gut has far-reaching health implications, affecting immune system development^2^, asthma and allergy^3–5^, obesity and rapid growth^6–9^, and cognitive development^10^. While the exact mechanisms by which the gut microbiome impacts human health are still being characterized, gut bacteria can impact host physiological systems through the production of metabolites that can have systemic health effects^11–13^. High resolution metabolomics is an emerging, analytical, omics-based technology which offers global detection and characterization of the human metabolome, affording insights into how the metabolome interacts with both exogenous and endogenous exposures, and downstream health implications.

To date, several studies have examined the impact of mode of delivery, antibiotic usage, and early life feeding practices on the infant fecal metabolome. For example, 6-week old infants born vaginally compared to caesarian section (CS) were shown to have enriched metabolic pathways related to carbohydrate metabolism including glycolysis/gluconeogenesis and glyoxylate and dicarboxylate metabolism^14^. Amino acid metabolism has also been shown to be pronounced in infants exposed to antibiotics during the first weeks of life^16,17^ as well as among formula-fed infants, compared to breastfed infants^14^. Further, breastfed infants have different metabolic profiles compared to their formula-fed counterparts at 3-, 6-, and 9-months of age, including differences in butyric acid, d-sphingosine, betaine, and kynurenic acid^18^. However, few studies have characterized the fecal metabolome using frequent repeated sampling during the first 2 years of life. This information is critical to understanding immune system development as infants increase food diversity and gain mobility.

While prior research offers important insight into the development of the fecal metabolome, previous longitudinal studies followed infants only through the first year of life^15–20^, and none have been conducted in a Latino cohort. The aim of this study was to contribute to the existing literature by systematically describing the development of the infant fecal metabolome during the first 2 years of life. Specifically, we examined the fecal metabolome at 1-, 6-, 12-, 18-, and 24-months of age in 101 Latino infants from the Southern California Mother’s Milk Study – a well-established cohort that was recruited based on an intention to breastfeed for at least three months. As a secondary aim, we sought to assess which factors were important predictors of overall fecal metabolites and fecal metabolomic pathways, including mode of delivery, antibiotic exposure, and infant age. Future work in this cohort will expand on this initial analysis to examine how breastfeeding, formula feeding, solid food introduction, and environmental exposures impact fecal metabolome throughout the first 2 years of life.

## Results

### Study population characteristics

General population characteristics are shown in Table 1. At the 1-month postpartum visit, mothers were 29 ± 6 years old (18–45), and most had a body mass index (BMI) in the overweight (33%) or obese (40%) category. The average infant age at fecal metabolome assessment was 1.1, 6.2, 12.3, 18.3, and 25.1 months, respectively, at planned 1-, 6-, 12-, 18-, and 24-month collection^21^. Half of the infants were female, 74% were born vaginally, and 10.9% received antibiotics in the first 2 years of life. Most (97%) were breastfed at 1-month, and 41% were breastfed at 24-months of age.

**Table 1:** Characteristics of 101 mother-infant dyads from the Southern California Mother’s Milk Study, 2016–2019. Data reported are mean and standard deviation (SD) unless otherwise noted.

	Mean ± SD or N, % N = 101
Maternal characteristics	
Age (years) at 1-month postpartum visit	29 ± 6
Socioeconomic status (SES)^a^	27 ± 12
Pre-pregnancy BMI (kg/m^2^)	28.7 ± 6.0
Infant characteristics	
Age (days) at 1-month postpartum visit	32.8 ± 3.1
Sex (Female, Male, %Female)	51, 50, 50.5%
Age of solid foods (months)^b^	5.9 ± 1.7
Antibiotics (Yes, No, %Yes)	11, 90, 10.9%
Birth mode (Vaginal, C-section, %Vaginal)	75, 26, 74.3%
Birth weight (kg)	3.4 ± 0.4
Birth length (cm)	50.4 ± 2.5
Gestational age	
Early (< 38 weeks gestation)	26 (25.7%)
On time (38–42 weeks gestation)	54 (53.5%)
Late (> 42 weeks gestation)	21 (20.8%)

^a^ Median replacement was performed for missing values (N = 2).

^b^ N = 100.

SES, socioeconomic status; BMI, body mass index.

### Temporal trends in fecal metabolites

**Figure 1:**
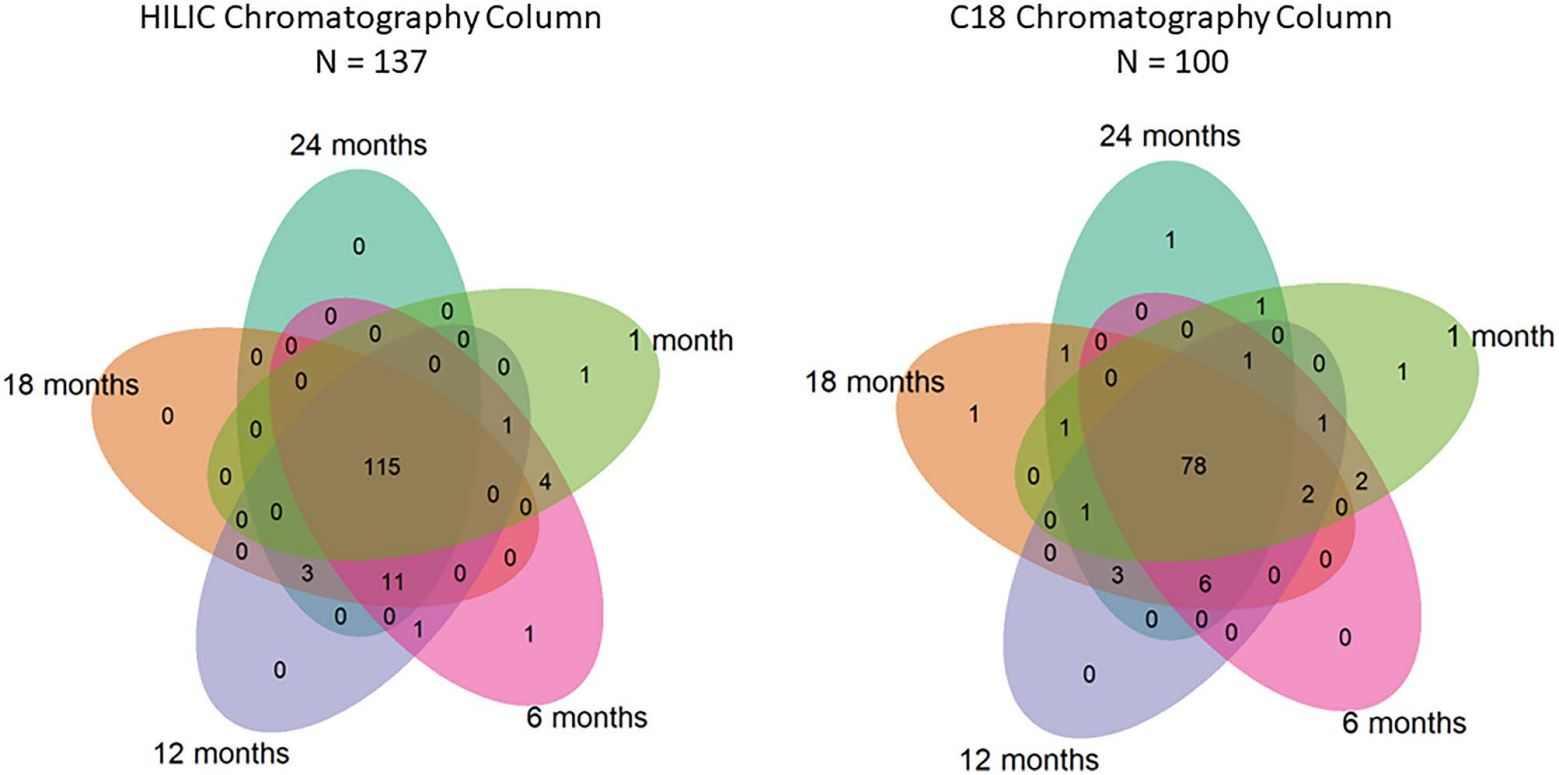
Confirmed metabolites observed longitudinally in at least 50% of samples during the first 2 years of life in the HILIC (left) and C18 (right) chromatography columns.

Overall, there were 11,345 metabolic features extracted from the HILIC and 8,609 in the C18 chromatography after removal of features that were present in less than 10% of samples. From this, we confirmed the chemical identities of 143 unique metabolites from HILIC chromatography and 104 metabolites from C18 chromatography with Level 1 evidence (i.e., features whose *m/z* and retention time could be matched to authentic standards with MS/MS under identical conditions). Among the confirmed metabolites, many were highly conserved over time. For instance, 115/143 metabolites in the HILIC column were observed in at least 50% of samples at each timepoint, as were 78/104 metabolites in the C18 column (Fig. 1). As a sensitivity analysis, we additionally explored metabolites that were present in 25% and 75% of samples (Supplemental Fig. 1 and Supplemental Fig. 2). We found that there were 135 and 97 confirmed metabolites that were present in at least 25% of samples at each timepoint in the HILIC and C18 column, respectively. There were 72 and 50 metabolites present in 75% or more of samples at every timepoint in the HILIC and C18 columns, respectively.

**Figure 2:**
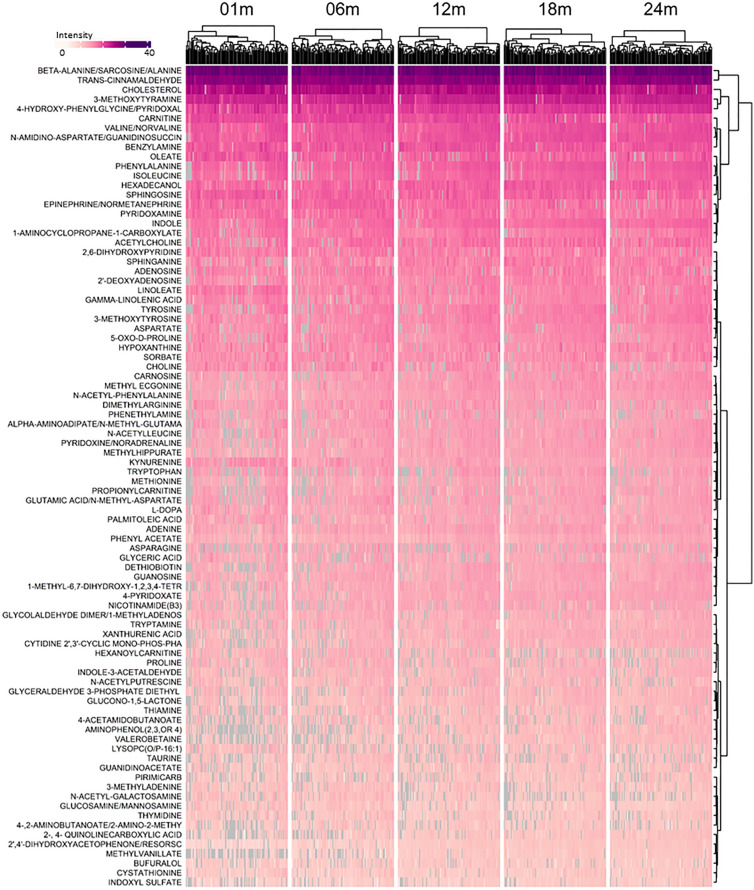
Longitudinal patterns of metabolite intensity of confirmed fecal metabolites detected in the HILIC chromatography column during the first 2 years of life. Intensity presented is standardized by dividing by intensity standard deviation, and ranges from 3.1 to 32.4, with dark purple representing the highest intensity and white representing the lowest intensity. Grey indicates a missing value. Metabolites detected in at least 80% of samples were included. Observations are grouped by visit, with the age in days corresponding with each visit as follows: 01 m (25–46 days), 06 m (164–219 days), 12 m (351–429 days), 18 m (517–582 days), and 24 m (709–916 days).

**Figure 3:**
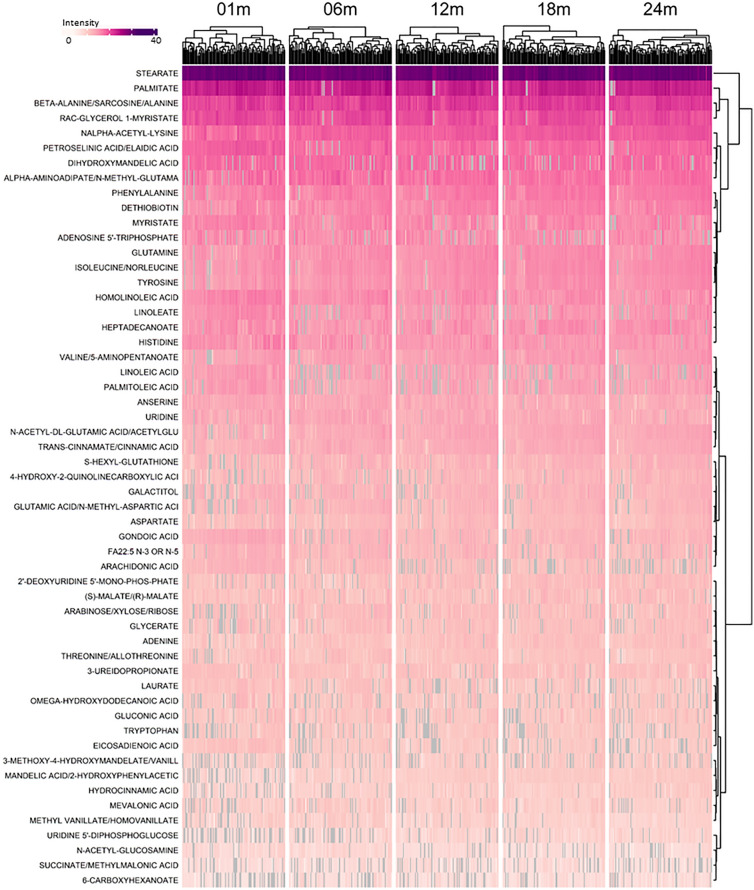
Longitudinal patterns of metabolite intensity of confirmed fecal metabolites detected in the C18 chromatography column during the first 2 years of life. Intensity presented is standardized by dividing by intensity standard deviation, and ranges from 4.7 to 37.6, with dark purple representing the highest intensity and white representing the lowest intensity. Grey indicates a missing value. Metabolites detected in at least 80% of samples were included. Observations are grouped by visit, with the age in days corresponding with each visit as follows: 01 m (25–46 days), 06 m (164–219 days), 12 m (351–429 days), 18 m (517–582 days), and 24 m (709–916 days).

Observed metabolites included (Figs. 2 and 3) amino acids (e.g., tyrosine, tryptophan, methionine, isoleucine, aspartate, proline), amino acid metabolites (e.g., phenylacetate, oxoproline, taurine, cystathionine), purines (adenine, guanine, hypoxanthine), pyrimidine (thymine) and vitamin metabolites [thiamine (B1), nicotinamide (B3), pyridoxine (B6), pyridoxate, alpha-tocopherol (E), dethiobiotin). Lipid metabolites included sphingosine, sphinganine, choline, carnitine, cholesterol, free fatty acids (e.g., linoleate, oleate) and lysophosphatidyl choline. Microbial metabolites with known adverse activities (indoxyl sulphate, valerobetaine) were present, and dietary (cinnamaldehyde) and environmental chemicals (pirimicarb) were also detected.

### Changes in fecal metabolites were largely driven by age and intra-infant variability

Given that the intensities of many confirmed metabolites changed during the first 24-months of life (Fig. 2 and 3), we performed non-parametric univariate permutational multivariate analysis of variance tests (PERMANOVA) to assess which variables explained the most variance in overall metabolite composition. We found that metabolic variation was largely driven by intra-individual variability such that 29.2% (*P* = 0.001) of the variability in confirmed metabolites in the HILIC column, and 30.0% (*P* = 0.001) of the variability in confirmed metabolites in the C18 column could be attributed to the infant providing the sample.

**Figure 4:**
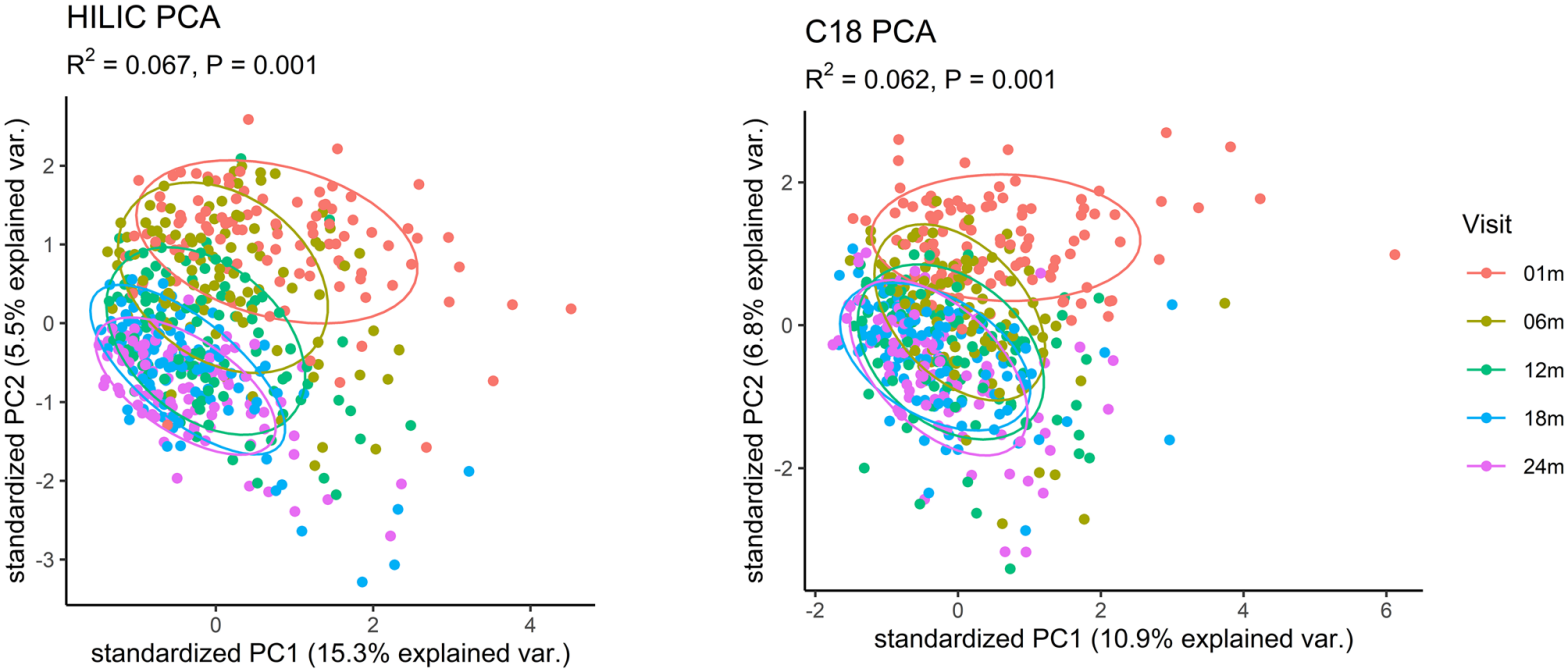
Ordination plot of principal components 1 and 2 for confirmed metabolites detected in HILIC (left) and C18 (right) by infant age in days. R^2^- and *P*- values calculated using Permutational Multivariate Analysis of Variance, with age in days as the explanatory variable. Points are colored by visit, with the age in days corresponding with each visit as follows: 01 m (25–46 days), 06 m (164–219 days), 12 m (351–429 days), 18 m (517–582 days), and 24 m (709–916 days).

In addition to individual variability, we also examined the impact of infant sex, antibiotic exposure (i.e., having received any antibiotics since birth), and infant age in days on overall profiles of fecal metabolites. Infant sex and antibiotic exposure were not important predictors of metabolites in the HILIC column, with sex explaining just 0.3% of variability (*P* = 0.1) and antibiotic exposure explaining 0.4% of variability (*P* = 0.02). Similarly, infant sex explained 0.2% of variability in the metabolites in the C18 column (*P* = 0.3) and antibiotic exposure explained 0.3% of variability (*P* = 0.1). Despite this, infant age explained much more of the variability in fecal metabolites where 6.7% (*P* = 0.001) and 6.2% (*P* = 0.001) of the variability in confirmed metabolites in the HILIC and C18 columns were explained by infant age (Fig. 4). Indeed, after adjustment for multiple testing using the Benjamini-Hochberg (BH) procedure, we found there were 87 confirmed metabolites in the HILIC chromatography column and 77 in the C18 chromatography column which were significantly associated with infant age in days (*P*_BH_ < 0.05). Table 2 summarizes the top 10 most significant results for each chromatography column. These showed increases in some diet-derived metabolites (methyl vanillate, hydrocinnamic acid) and decreases in free fatty acids (arachidonate, laurate, homolinoleic acid). A complete list of statistically significant results can be found in Supplemental Table 2.

**Table 2:** Top ten confirmed metabolites detected by HILIC and C18 chromatography columns that were most significantly associated with infant age, based on the results of linear mixed effects models with random intercepts for individual to account for repeated measures. Results from these models were adjusted using the Benjamini–Hochberg (BH) procedure. Overall, there were 87 metabolites significantly associated with infant age in days after adjustment for multiple testing in the HILIC chromatography column and 77 in the C18 chromatography column.

HILIC chromatography column			C18 chromatography column		
Metabolite	Direction	P_BH_	Metabolite	Direction	P_BH_
4-Pyridoxate	↑	1.2 × 10^–47^	Arachidonic Acid	↓	2.3 × 10^–34^
Dihydroxyacetophenone/ Resorscinol monoacetate/ Methylparaben	↑	5.7 × 10^–55^	Docosahexaenoic acid	↓	1.8 × 10^–34^
Hexadecanol	↑	4.2 × 10^–18^	Eicosadienoic acid	↓	3.5 × 10^–20^
Kynurenine	↓	1.3 × 10^–26^	Gondoic acid	↓	3.1 × 10^–17^
Methylnicotinium*	↑	8.3 × 10^–27^	Homolinoleic acid	↓	2.3 × 10^–18^
Methylhippurate	↑	1.0 × 10^–17^	Hydrocinnamic acid	↑	1.3 × 10^–18^
Methyl vanillate	↑	2.5 × 10^–21^	Hypoxanthine	↓	1.0 × 10^–15^
*N*-Acetyl-phenylalanine	↑	3.3 × 10^–19^	Laurate	↓	1.6 × 10^–16^
Valerobetaine	↑	2.7 × 10^–35^	Mandelic acid/2-,3-Hydroxyphenylacetic acid	↑	3.0 × 10^–41^
Xanthurenic acid	↑	3.6 × 10^–22^	*N*-alpha-acetyl-L-lysine	↑	3.0 × 10^–27^

*This compound was unidentified but is an accurate mass match for methylnicotinium.

**Figure 5:**
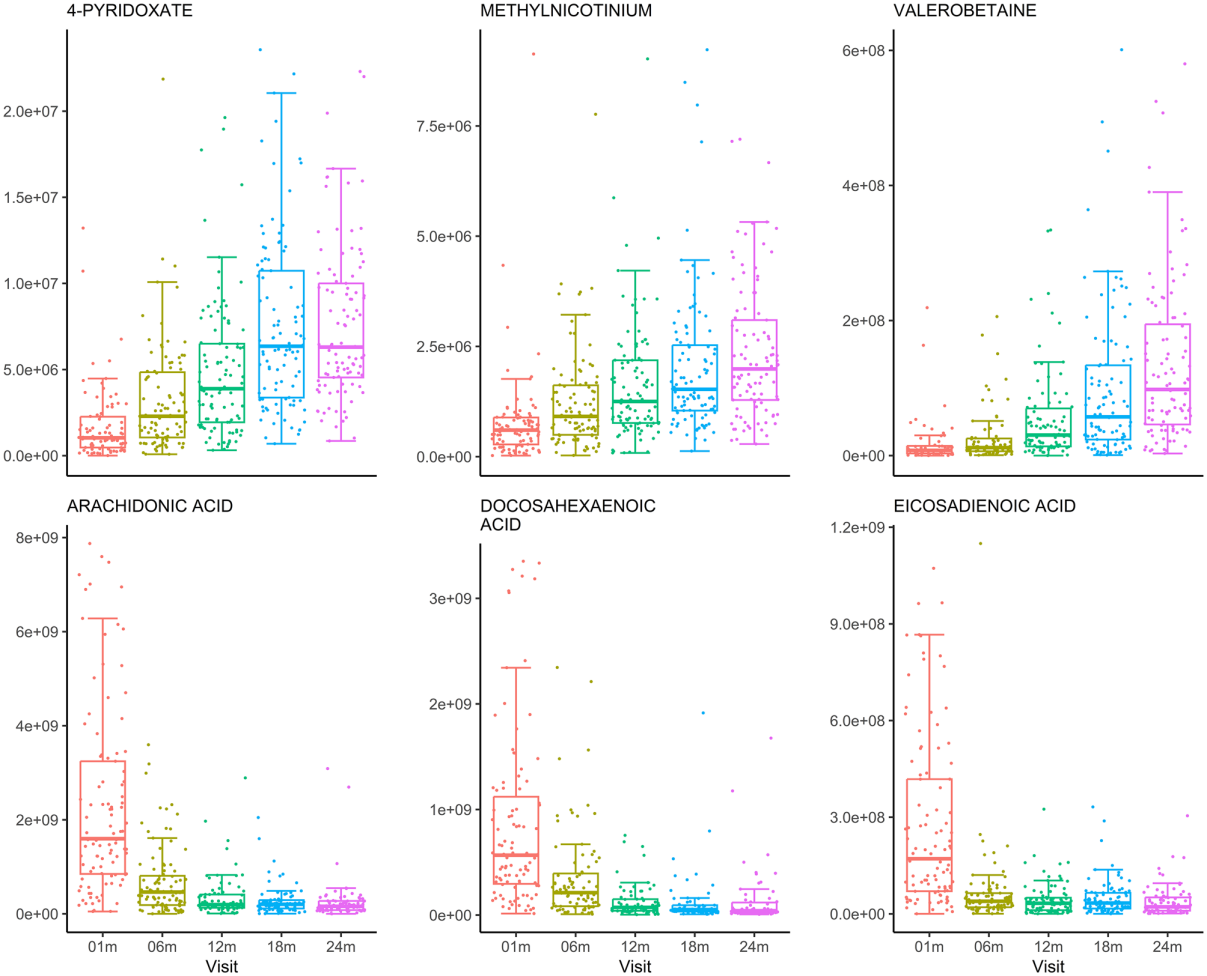
Visual representation of the intensity of selected confirmed metabolites detected in the HILIC (top) and C18 (bottom) chromatography columns which varied most significantly with infant age. Observations are grouped by visit, with the age in days corresponding with each visit as follows: 01 m (25–46), 06 m (164–219), 12 m (351–429), 18 m (517–582), and 24 m (709–916). The metabolite labeled methylnicotinium was unidentified but was an accurate mass match for methylnicotinium.

As shown in Fig. 5, we examined selected metabolites that were most significantly associated with infant age in days in the HILIC and C18 chromatography columns. Mean intensity of valerobetaine, a microbiome-derived metabolite associated with increased adiposity^22^, increased by 588% between 1- and 24-months of age. Other confirmed metabolites in the HILIC column that decreased with infant age included kynurenine, a metabolite associated with immune cell function, whose mean intensity decreased by 79.1%. In the C18 column, mean intensity of the polyunsaturated fatty acids arachidonic acid and eicosadienoic acid decreased with infant age (an 88.5% and 85.5% decrease, respectively). In addition to examining the level of intensity over time, we also visualized the prevalence of each metabolite that was most significantly associated with infant age (Supplemental Fig. 3). While most of these metabolites were detected with high prevalence across all timepoints, valerobetaine prevalence increased dramatically over time and prevalence of arachidonic and docosahexaenoic acid decreased precipitously between 1- and 24-months.

### Amino acid, bile acid, and carbohydrate metabolism pathways were enriched with infant age

**Figure 6:**
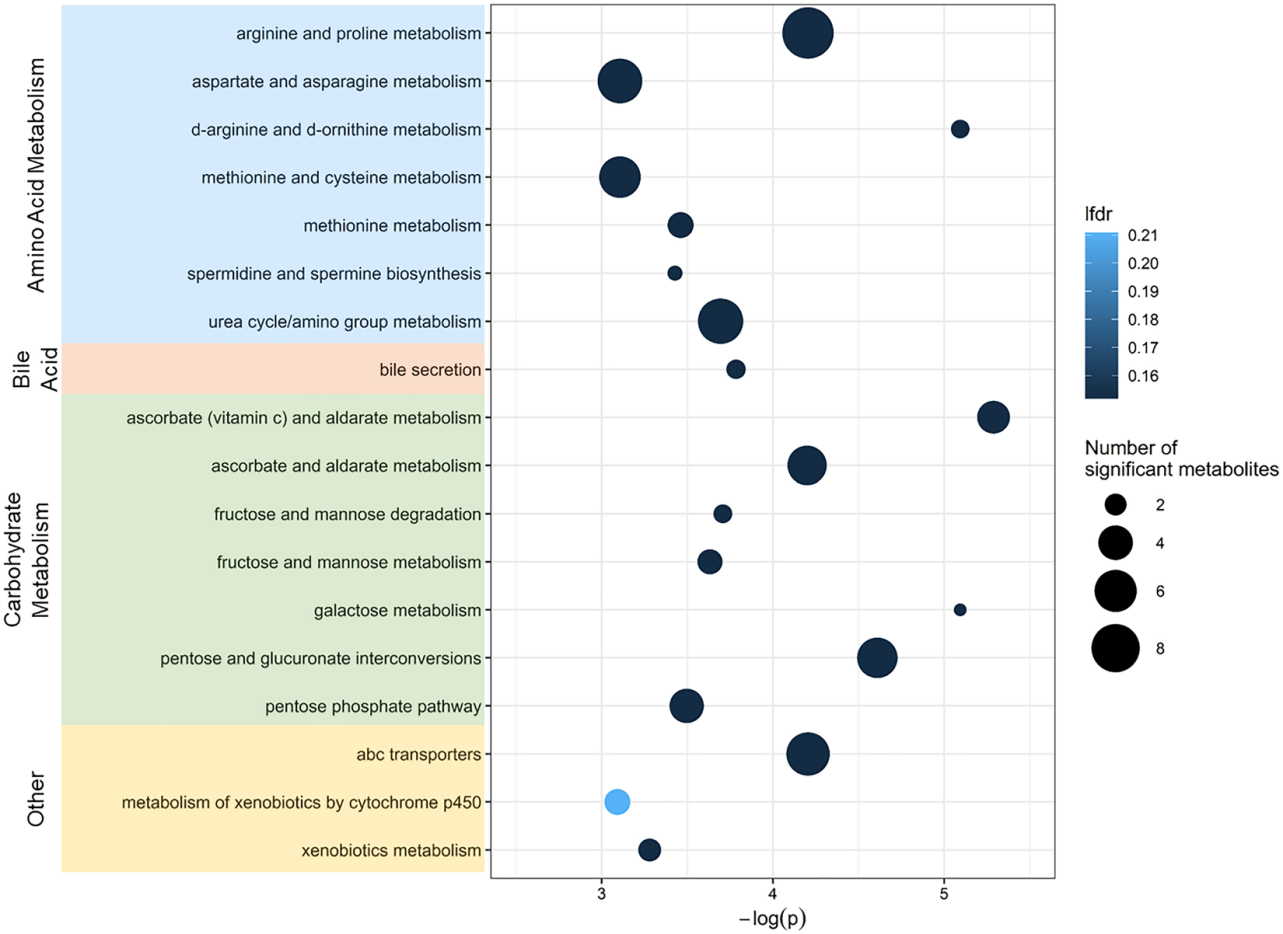
Overview of enriched pathways by confirmed HILIC and C18 chromatography column metabolites that were significantly associated with infant age, identified using Metapone. Pathways with P ≥ 0.05 and weighted number of significant metabolites ≥ 1.5 are included. Point size indicates the number of significant metabolites identified within each pathway and point color indicates significance after adjustment for multiple testing via conditional local false discovery rate (lfdr). Pathways are grouped by super pathway (amino acid metabolism, bile acid, carbohydrate metabolism, lipid, and other), indicated by color.

Results from the linear mixed effects models were used to perform pathway enrichment analyses using Metapone^23^. There were 18 metabolic pathways significantly associated with infant age (*P* ≤ 0.05) and with weighted number of significant metabolites ≥ 1.5. Pathways associated with infant age are summarized in Fig. 6**.** Of the pathways associated with infant age, 7 pathways were related to amino acid metabolism, including both non-essential and essential amino acid pathways and the urea cycle. These pathways are linked to many of the metabolites in Table 2 (e.g., pyridoxine is the vitamin supporting amino acid nitrogen elimination through the urea cycle). Two pathways containing methionine are connected to individual metabolites described above (e.g., taurine), and to the bile acid pathway and the spermidine/spermine pathway. Seven pathways were related to carbohydrate metabolism, and one was related to bile acid metabolism. Other significant pathways included metabolism of xenobiotics and membrane transport (ATP-binding cassette transporters).

## Discussion

This study provides important new information concerning the longitudinal development of the fecal metabolome in Latino infants from 1 to 24 months, a time period with far-reaching health implications for immune system development^2^, asthma and allergy^3–5^, obesity and growth^6–9^, and cognitive development^10^. At least 50% prevalence at each timepoint was observed for about 200 confirmed metabolites, indicating that the fecal metabolome was relatively stable. Variability was primarily explained by the individual providing the sample as well as infant age. Metabolites associated with infant age included amino acid metabolites, such as kynurenine, and polyunsaturated fatty acids, arachidonic and docosahexaenoic acid. Of potential importance relative to weight gain and obesity, valerobetaine increased substantially over the first 24 months. Valerobetaine is a metabolite produced by the intestinal microbiome which was recently found to inhibit fatty acid oxidation by mitochondria^22^. In mice, valerobetaine causes increased adiposity and in humans, valerobetaine is associated with high BMI and fatty liver^22^. The latter association may be especially relevant to Latino health because adolescent Latino children have increased risk of non-alcoholic fatty liver disease (NAFLD)^24,25^. Other metabolites whose intensity was significantly associated with infant age were also associated with pathways related to amino acid metabolism, carbohydrate metabolism, bile acid metabolism, and lipid metabolism.

A previous study on preterm infants found that intra-individual variability explained 43% of variation in fecal metabolites during the first months of life; whereas, health outcomes (sepsis and necrotizing enterocolitis), delivery mode, and antibiotics were not significantly associated with metabolomic variation^26^. Several of the metabolites that we found to be related to infant age have also been identified previously. For example, we found that uracil, aspartate, methionine, tyrosine, phenylalanine, and valine were significantly increased with infant age, while glucose and fucose metabolites were significantly decreased. Interestingly, previous work has found these same metabolites to be associated with either formula or breast feeding^14^. We also observed that methyl vanillate, which is a compound identified in cow’s milk^27^ and also produced by Brewer’s yeast (Saccharomyces cerevisiae)^28^, significantly increased with infant age. Given this, the changes that we observed in metabolite intensity over time may reflect increased formula or cow’s milk consumption, increased consumption of solid foods, and/or decreased breastfeeding or weaning as infants age. Several metabolites that changed significantly over time in our study are found in breast milk and infant formula, including kynurenine, which is present in both breast milk and formula, but is found in higher amounts in formula compared to breast milk^29^. Arachidonic acid and docosahexaenoic acid (DHA) are both long-chain polyunsaturated fatty acids that are found in breast milk^30^. Infant formulas are also supplemented with DHA^31^. These observations further bolster the hypothesis that shifts in the early-life fecal metabolome are driven largely by dietary alterations during the first 2 years of life. Given these observations, future work in this cohort will explore the associations between the fecal metabolome with early life feeding practices, the infant gut microbiota, and adiposity.

In this study, we found that pathways related to amino acid metabolism, carbohydrate metabolism, and bile acid metabolism were associated with increased infant age. Interestingly, several of these pathways have also been linked with infant breast or formula feeding^14^. For example, we observed that seven amino acid pathways increased with infant age, one of which (arginine and proline metabolism) has been shown to be enriched in the fecal metabolome of formula-fed infants compared to breastfed infants at 6-weeks of age^14^. We also found seven carbohydrate metabolism pathways enriched with infant age, two of which (galactose metabolism and fructose and mannose metabolism) were enriched in breastfed infants, compared to formula-fed infants^14^.

Pathways that we identified in the current analysis have also been linked with antibiotic exposure and mode of delivery in other fecal metabolomics studies that investigated infants. For example, amino acid metabolism has previously been shown to be enriched in infants exposed to antibiotics during the first weeks of life^16,17^. We also found metabolites linked to ABC transporters were enriched with increasing infant age. Previous studies have observed fecal metabolome enrichment of this pathway among infants aged approximately 15-weeks and born cesarean-section, compared to vaginally-born infants^15^ and among preterm infants with antibiotic exposure during the first 14 days of life, compared to preterm infants who did not receive antibiotics^16^. Lastly, one carbohydrate metabolism pathway (galactose metabolism), which we found was enriched with infant age, has previously been shown to be enriched in 6-week old infants who were born by cesarean section^14^.

While this study had several strengths, including repeated sampling and comprehensive metabolic profiling of the infant fecal metabolome in a well-established cohort of infants, some limitations are worth noting. First, stool was collected using OMNIGene GUT kits, which may have decreased the number of identified metabolites^33^. However, previous studies have found that a much higher proportion of variability in the fecal metabolome is attributable to individual, compared with collection method^32^. Given the young age of our participants, we were unable to collect blood samples, which limited our ability to examine the circulating metabolome. Therefore, future studies should incorporate fecal metagenomics and serum metabolomics since gut bacteria likely play an important role in shaping the fecal metabolome. Additionally, as this study was focused on metabolites that were identified with Level 1 evidence, we largely characterized patterns in a relatively limited number of endogenous metabolites over the first 2 years of life. This study was also conducted in an exclusively Latino cohort with exclusions such as preterm birth or low birth weight, cigarette smoking or recreational drug use, which may limit the generalizability of these findings to the broader population. In particular, given that our cohort was comprised of Latinos we were unable to assess whether race/ethnicity is an important predictor of the fecal metabolome. Future work is needed to replicate these findings in other populations. Nevertheless, we detected numerous metabolites that were previously observed in other infant populations. Lastly, exploring feeding practices in this cohort is complex because mothers were recruited based on an intention to breastfeed. Furthermore, while weaning may impact the fecal metabolome, we were unable to address weaning in this study due to incomplete information. Therefore, while examining infant feeding was beyond the scope of the current study, future work will be needed to tease apart the relative intake of formula and breast milk over time and characterize how breastfeeding, formula feeding, and solid food introduction impact fecal metabolome throughout the first 2 years of life.

## Conclusions

This study characterizes the development of the infant fecal metabolome during the first 2 years of life. Overall, we found evidence that the metabolome was relatively stable across time and highly personalized, with metabolic profiles being largely driven by intra-individual variability. We also identified several novel metabolites, such as valerobetaine, and pathways, including amino acid biosynthesis, which were significantly associated with infant age.

## Methods

### Study population

The Southern California Mother’s Milk Study is an ongoing, longitudinal cohort of 219 Latino mother-infant pairs who were recruited between 2016 and 2019 from Los Angeles County maternity clinics, which has been previously described^21^. Participants were eligible to participate if they were ≥ 18 years old at time of delivery; had a healthy, singleton birth; enrolled in the study by 1-month postpartum; and could read at a 5^th^ grade level in either Spanish or English. Potential participants were excluded if they had any diagnoses known to impact mental/physical health, nutritional status, or metabolism; were currently using tobacco or recreational drugs; had infants who were pre-term or low birth weight; or had infants with clinically diagnosed fetal abnormalities. The Institutional Review Boards of the University of Southern California, Children’s Hospital Los Angeles, and the University of Colorado Boulder approved of the study procedures and all research was performed in accordance with the relevant guidelines and regulations. Written informed consent was obtained from participants at time of enrollment.

### Study design

Participants were enrolled by 1-month postpartum and attended follow-up visits at 6-, 12-, 18-, and 24-months postpartum. 219 mother-infant dyads were initially enrolled in the Mother’s Milk cohort. As previously reported^34^, the primary aim of the Mother’s Milk Study was to assess the impact of sugars and human milk oligosaccharides on the infant microbiome and obesity. Briefly, socioeconomic status was estimated using a modified Hollingshead index, as previously described^21,35^. Maternal self-report was used to classify infants’ gestational age as early (< 38 weeks gestation), on time (38–42 weeks gestation), and late (> 42 weeks gestation). Questionnaires were used to determine birth mode, infant antibiotic exposure, and infant feeding practices. Additional funding supported the analysis of 600 fecal metabolomics samples in this cohort and a subset of 127 participants were selected to undergo fecal metabolomics analysis, to maximize the number of participants with repeated fecal metabolome samples. 101 infants had complete fecal metabolomics samples at all 5 visits and were included in this analysis. Those individuals that were excluded due to missing data did not differ significantly from those who were included in the analysis (Supplemental Table 1).

### High-resolution metabolomics

OmniGene GUT kits were used to collect infant stool samples at 1-, 6-, 12-, 18-, and 24- months of age. Untargeted high-resolution metabolomics analysis was carried out by the Emory Clinical Biomarkers Laboratory, as previously described^36,37^. To precipitate proteins, stool samples were first added to ice-cold acetonitrile. Samples were then kept on ice for 30 min, centrifuged for 10 min at 14,000 g, and kept at 4 °C until analysis. Extractants were examined in triplicate using high-resolution mass spectrometry (LC-HRMS) (Dionex Ultimate 3000, Thermo Scientific Orbitrap Fusion).

### Instrumentation and analytical conditions

Hydrophilic interaction liquid chromatography (HILIC) (Waters XBridge BEH Amide XP HILIC column; 2.1 × 50 mm^2^, 2.6 μm particle size) with positive electrospray ionization (ESI) and reverse phase (C18) chromatography (Higgins Targa C18 2.1 × 50 mm^2^, 3 μm particle size) with negative ESI were used. HILIC analyte separation was conducted using water, acetonitrile, and 2% formic acid mobile phases following the subsequent gradient elution. The initial 1.5-min period consisted of 22.5% water, 75% acetonitrile, and 2.5% formic acid followed by a linear increase to 75% water, 22.5% acetonitrile, and 2.5% formic acid at 4 min, followed by a final hold for 1 min. Analyte separation for the C18 chromatography column was conducted using water, acetonitrile, and 10 mM ammonium acetate mobile phases under the following gradient elution. The initial 1-min period consisted of 60% water, 35% acetonitrile, and 5% ammonium acetate followed by a linear increase to 0% water, 95% acetonitrile, and 5% ammonium acetate at 3 min with a final hold for the last 2 min. Mobile phase flow rate was 0.35 mL/min for the first minute and was increased to 0.4 mL/min for the last 4 min for both the HILIC and C18 chromatography columns. LC-HRMS was run in full scan mode, with 120 k resolution and had a range of mass-to-charge ratio (*m/z*) from 85 to 1,275. Tuning parameters for sheath gas were 45 (arbitrary units) for positive ESI and 30 for negative ESI. For positive ESI, auxiliary gas was set to 25 (arbitrary units) and spray voltage was set at 3.5 kV, and for negative ESI, auxiliary gas was set to 5 and spray voltage was set to − 3.0 kV. Internal standards included pooled stool and standard reference materials for human metabolites in stool. These internal standards were added at the beginning and end of each 20-sample batch for quality control and standardization.

### Metabolite confidence and identification

Data from positive and negative ion modes were analyzed separately, and raw files were converted to the .mzXML format. Then, metabolomic signals (i.e., metabolic features) were extracted and aligned using apLCMS with modification of xMSanalyzer for quality control and reduction of batch effects following instrument analysis^38,39^. The coefficients of variation (CV) of metabolites were assessed as part of quality control. Metabolites whose intensity had CV > 30% were removed and intensities of metabolic features were averaged across triplicates. Metabolic features which were detected in < 10% of samples were excluded. Outliers were assessed visually using principal component analysis (PCA) of the log_2_ transformed metabolite intensities. In a sensitivity analysis, samples with PCA score was > 3 standard deviations for PC 1 or PC 2 were removed (not shown). However, there were no important differences in results, so these observations were not removed. Metabolomic features were then annotated and confirmed using the Metabolomics Standards initiative criteria^40^. Level 1 confidence was assigned to features whose *m/z* and retention time matched the authentic standards analyzed with MS/MS under identical conditions (within 10 ppm and 50 s).

### Statistical analysis

Descriptive statistics for key variables were performed on the full analytic data set. We used the VennDiagram package in R to visualize how many metabolites were present in 25%, 50%, and 75% of samples at each visit^41^. Heatmaps to visualize log_2_ transformed metabolite intensities over time were generated using the ComplexHeatmap package in R^42^. Next, we performed PCA on the log_2_ transformed metabolite intensities to visualize overall metabolite profiles over time. We used permutational multivariate ANOVA (PERMANOVA) tests to explore how overall fecal metabolite intensities changed over time, and in relation to individual, infant sex, and antibiotic exposure using the adonis2 function implemented by the vegan package in R, using Euclidian distance (permutations = 1000)^43^. Linear mixed effects models were then used to estimate the relationship between the log_2_ transformed intensity of each confirmed metabolite and infant age in days using the lme4 package in R^44^. Models included random intercepts to account for repeated measures and were adjusted for multiple testing using the Benjamini–Hochberg procedure^45^. Boxplots were used to visualize the intensity of 6 selected confirmed metabolites associated with infant age in days in the HILIC and C18 chromatography columns by visit. Finally, we used the Metapone package in R^23^ to jointly assess which metabolic pathways were associated with infant age in days, with combined linear mixed effects model results generated for the HILIC and C18 chromatography columns, using a weighted gene set enrichment analysis (GSEA) modified to untargeted metabolomics data. Briefly, Metapone employs established online MS databases of metabolites to putatively annotate metabolic features, incorporates a weight to limit the influence of single features that are annotated to multiple metabolites, and finally applies the modified GSEA test to focus on groups of metabolic features playing a role in the same biological pathways^23^. Metapone uses local false discovery rate (lfdr) to adjust for multiple testing, which is a Bayesian approach with minimal a priori assumptions and which does not rely on the assumption that individual statistical tests are independent^46^.

## Supplementary Information

Supplementary Information.

Supplementary Table 2.

## Data Availability

Data cannot be shared publicly because they include potentially identifying information on human subjects. The data that support the findings of this study are available upon reasonable request from the corresponding author, TLA.
