# Supplementary material for: Longitudinal profiles of the fecal metabolome during the first 2 years of life
Source: Sci Rep. 2023 Feb 2;13:1886. doi: 10.1038/s41598-023-28862-z (PMC9895434; doi:10.1038/s41598-023-28862-z)
Supplement: Supplementary file 1 — Supplementary Information. [file 41598_2023_28862_MOESM1_ESM.docx]

**Supplemental Table 1.** Mother-infant dyads who were included in the present analysis did not differ significantly at baseline from those who were excluded due to missing metabolomics data. Baseline (1-month) characteristics of 219 Latino mother-infant dyads from the Southern California Mother’s Milk Study, stratified on inclusion into the current analysis. For continuous variables, independent t-test were used to test for differences between those who were included in the analysis and those who were excluded due to missing metabolomics data. For categorical variables, Chi-square test were used to test for differences between those who were included compared to those who were excluded.

|  | **Included in Analysis**  **Mean ±** **SD or N, %**  **N = 101** | **Excluded from analysis**  **Mean ±** **SD or N, %**  **N = 118** | **P-value** |
| --- | --- | --- | --- |
| **Maternal Characteristics** | | | |
| Age (years) at 1-month postpartum visit | 29 ± 6 | 29 ± 6 | 0.97 |
| Socioeconomic status (SES) | 27 ± 12 | 26 ± 12 | 0.46 |
| Pre-pregnancy BMI (kg/m)^2^ | 28.7 ± 6.0 | 28.4 ± 5.9 | 0.72 |
| **Infant Characteristics** | | | |
| Age (days) at 1-month postpartum visit | 32.8 ± 3.1 | 32.4 ± 5.6 | 0.60 |
| Sex (Female, Male, %Female) | 51, 50, 50.5% | 66,52, 55.6 | 0.54 |
| Age of solid foods (months) | 5.9 ± 1.7 | 5.7 ± 1.6 | 0.35 |
| Antibiotics (Yes, No, %Yes) | 11, 90, 10.9% | 9, 109, 8.3% | 0.69 |
| Birth mode (Vaginal, C-section, %Vaginal) | 75, 26, 74.3% | 91, 27, 77% | 0.99 |
| Birth weight (kg) | 3.4 ± 0.4 | 3.4 ± 0.4 | 0.92 |
| Birth length (cm) | 50.4 ± 2.5 | 50.5 ± 2.3 | 0.91 |
| Gestational age  Early (< 38 weeks gestation)  On time (38-42 weeks gestation)  Late (> 42 weeks gestation) | 26 (25.7%)  54 (53.5%)  21 (20.8%) | 22 (18.6%)  68 (57.6%)  28 (23.7%) | 0.44 |

**Supplemental Table 2.** All confirmed metabolites detected by HILIC and C18 chromatography columns that were significantly associated with infant age, based on the results of several linear mixed effects models estimating the relationship of metabolite intensity with infant age in days. Models included random intercepts for individuals to account for repeated measures. Results were adjusted for multiple testing using the Benjamini-Hochberg (BH) procedure.

**Supplemental Figure 1.** Confirmed metabolites observed longitudinally in at least 25% of samples during the first 2 years of life in the HILIC (left) and C18 (right) chromatography columns.


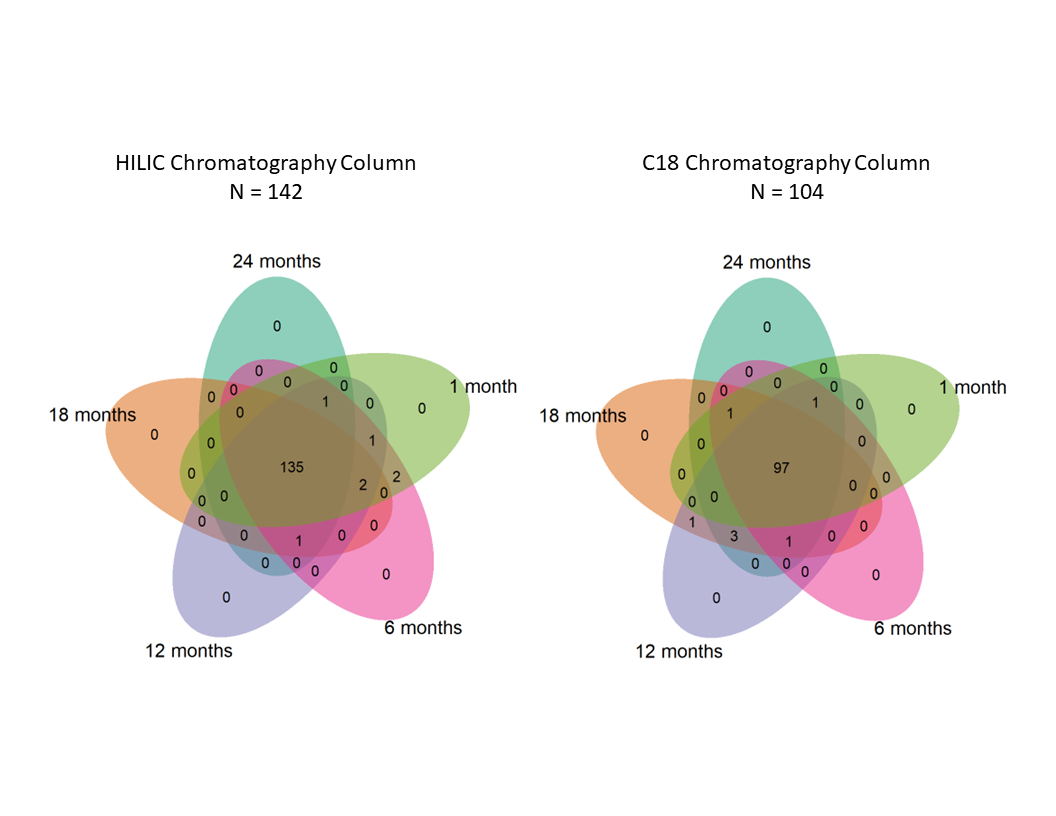


**Supplemental Figure 2.** Confirmed metabolites observed longitudinally in at least 75% of samples during the first 2 years of life in the HILIC (left) and C18 (right) chromatography columns.


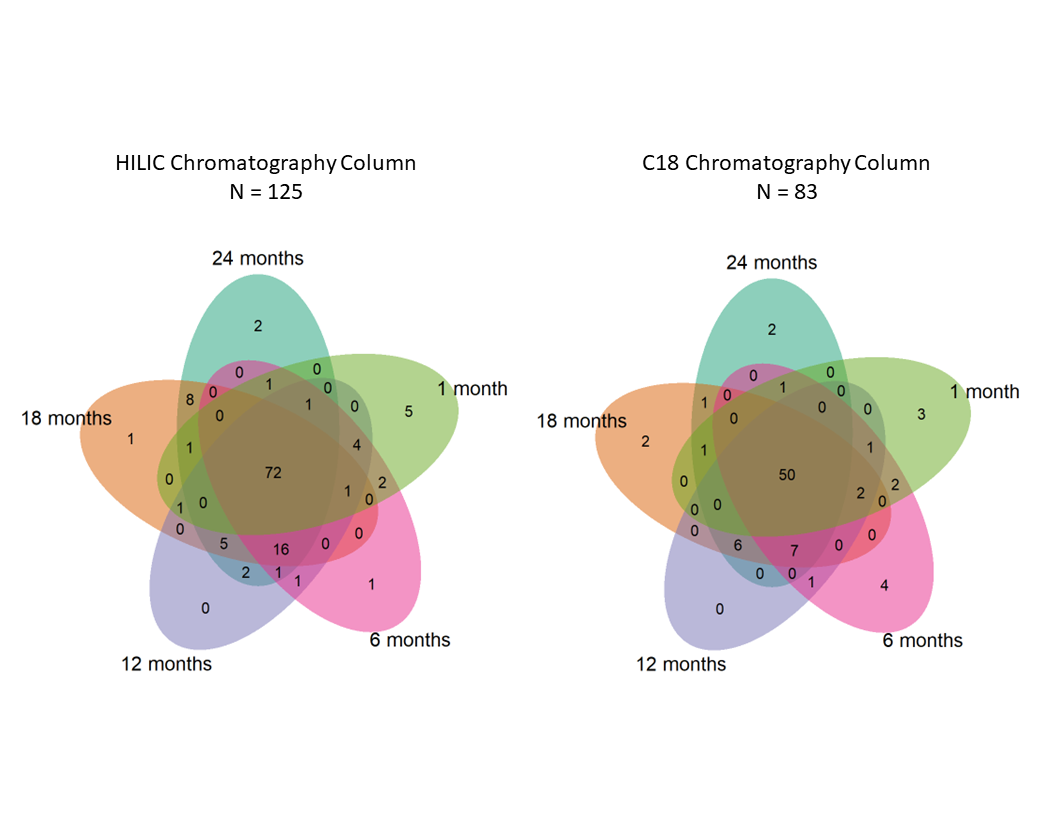


**Supplemental Figure 3.** Longitudinal prevalence patterns of the top 10 metabolites from the HILIC (top) and C18 (bottom) chromatography columns whose intenstiy was most significantly associated with infant age, based on the results of linear mixed effects models.
